# Primary health care data-based early warning system for dengue outbreaks: a nationwide case study in Brazil

**DOI:** 10.1016/j.lana.2025.101165

**Published:** 2025-07-04

**Authors:** Rejane Santos-Silva, Pilar Tavares Veras Florentino, Thiago Cerqueira-Silva, Vinicius de Araújo Oliveira, Juracy Bertoldo Junior, George C.G. Barbosa, Gerson O. Penna, Viviane S. Boaventura, Pablo I. Pereira Ramos, Manoel Barral-Netto, Izabel Marcilio

**Affiliations:** a Centro de Integração de Dados e Conhecimento para Saúde (Cidacs), Instituto Gonçalo Moniz, Fundação Oswaldo Cruz (Fiocruz Bahia), Salvador, Brazil; b Laboratório de Medicina e Saúde Pública de Precisão, Fundação Oswaldo Cruz, Salvador, Brazil; c Faculty of Epidemiology and Population Health, London School of Hygiene and Tropical Medicine, London, United Kingdom; d Faculdade de Medicina da Bahia, Universidade Federal da Bahia, Salvador, Brazil; e Núcleo de Medicina Tropical, Universidade de Brasília, Brasília, Brazil; f Escola de Governo Fiocruz Brasília, Fiocruz Brasília, Brasília, Brazil; g Escola Bahiana de Medicina e Saúde Pública, Salvador, Brazil

**Keywords:** Early warning system, Dengue, Arbovirus, Surveillance

## Abstract

**Background:**

Traditional surveillance presents limitations for early outbreak detection. Primary health care (PHC) administrative data applied to syndromic surveillance offers a cost-effective way to integrate early warning systems (EWS). We evaluate the potential of an EWS for dengue outbreaks using PHC data in Brazil.

**Methods:**

We applied the Early Aberration Reporting System (EARS-C1 and EARS-C2) to arbovirus-related PHC encounters from October 1, 2022, to March 1, 2024, to establish an EWS across 5570 municipalities. We assessed EWS timeliness, sensitivity, and positive predictive value (PPV) against fixed-incidence dengue outbreak thresholds.

**Findings:**

Arbovirus-related PHC encounters occurred in 5364 (96.3%) and dengue cases in 5269 (94.6%) Brazilian municipalities. PHC-based warnings anticipated 48.5% (100 cases/100,000 inhabitants), and 68.4% (300/100,000) of outbreaks detected by existing surveillance. Timeliness was higher in municipalities with over 100,000 inhabitants.

**Interpretation:**

The EARS algorithm applied to PHC data anticipated outbreaks up to four weeks before suspected case reporting. Its use of routine data ensures broader coverage and scalability. This study demonstrates the feasibility of integrating PHC data into an EWS for early dengue outbreak detection in Brazil.

**Funding:**

Rockefeller Foundation’s Health Initiative and 10.13039/501100006181Fundação de Amparo à Pesquisa do Estado da Bahia, Brazil.

**Research in context**

**Evidence before this study**

A significant increase in dengue incidence was observed in the past decade worldwide, and the disease is now endemic in more than 100 countries. In this context, an operational early warning system (EWS) is essential for strategic public health response. However, integrating an EWS into public health activities remains challenging. We searched the PubMed database for original articles published between January 2000 and December 2024 using the terms “early warning” AND “surveillance” AND (“dengue” OR “arbovirus”). Our search identified 194 articles, which were then screened by title and abstract. We identified studies evaluating the impact of climate and meteorological variables and other predictors on dengue incidence, epidemiological analysis of past outbreaks, studies on mosquito data and on methods for forecasting incidence and importation. Notably, our query retrieved only five studies focusing on the application of an EWS based on real case data, all conducted in Latin America and Southeast Asia, and all including climate and meteorological variables as predictors. However, we could not find studies using administrative data integrated into an automated EWS, despite the WHO research prioritization highlighting the importance of multidisciplinary data integration for improving epidemic detection.

**Added value of this study**

We report the development and implementation of an EWS for dengue outbreaks based on routinely collected administrative primary health care (PHC) data in Brazil. When comparing the performance of the PHC-based EWS to the passive surveillance system currently in practice, based on the notification of suspected cases by practitioners, we found that the EWS successfully identified between 76.6% and 85.5% of dengue outbreaks. Notably, it detected up to 69.3% of outbreaks up to four weeks earlier. Furthermore, the PHC-based EWS provided broader geographic coverage of epidemiological surveillance than systems relying on active case notification, with 95 more municipalities recording arbovirus-related PHC encounters than those reporting dengue cases to the surveillance system.

**Implications of all the available evidence**

Our findings demonstrate that the PHC-based EWS identified most outbreaks detected by the existing surveillance system while providing early warnings. This study demonstrates the feasibility of developing an operational EWS for dengue outbreaks based on routinely collected PHC administrative data to enhance the existing surveillance system. These findings are particularly relevant in the context of preparedness and response to health emergencies, given that data collection during an early outbreak phase is time-consuming, hindering the prompt deployment of countermeasures.

## Introduction

Dengue is a growing global public health concern. The disease is now endemic in over 100 countries, and the geographic range of the dengue vector distribution is expanding to regions of higher latitude and altitude.^1^, ^2^, ^3^ Over the past decade, there has been a substantial increase in dengue incidence globally, with over 14 million cases reported in 2024, including 7.3 million confirmed cases, and over 10,000 deaths.^3^ Dengue outbreaks negatively impact populations, especially the most vulnerable,^4^ can overwhelm health systems and severely affect economies,^1^ making an early warning system (EWS) for outbreaks essential to enable timely responses.^5^^,^^6^

Traditional dengue surveillance systems are generally passive and rely on the active reporting of suspected cases, with outbreak definition based on fixed-incidence thresholds. This often results in delays and underreporting, hence limiting early outbreak detection.^7^ Despite advances in dengue EWS, their integration into routine surveillance remains challenging. Key reasons for this include the complexity of models requiring highly skilled personnel, and the need for readily available data at spatial and temporal scales compatible with EWS analysis, such as climate, meteorological, and mosquito data.^8^, ^9^, ^10^

Conversely, routinely collected administrative data offers a practical and affordable approach to syndromic surveillance. This approach improves timeliness and sensitivity by leveraging information readily available before laboratory confirmation,^11^ enabling more efficient resource allocation.^12^ Moreover, the use of electronic health records facilitates implementing digital EWS based on routinely collected data, further addressing the timeliness and cost-effectiveness of surveillance systems, as it exempts the need for additional data collection efforts.^13^^,^^14^

In the context of establishing an EWS for dengue outbreaks, primary health care (PHC) data is particularly relevant. PHC services provide comprehensive coverage and granular information, often serving as the first point of contact for patients with health services. This is especially evident in Brazil, where the Unified Health System (Sistema Único de Saúde—SUS) provides universal, free-at-the-point-of-delivery health care to the entire population.^15^ Additionally, the effective management of SUS relies on diverse information systems, ultimately resulting in a wealth of administrative health datasets. This allows for the capture of timely, location-based data that is systematically and continuously collected, producing a rich source for real-time analysis. In this study, we evaluate the potential of an EWS based on syndromic surveillance using PHC data for the early detection of dengue outbreaks to enhance the existing dengue surveillance system in Brazil.

## Methods

### Study design

This was a retrospective, nationwide case study for assessing the capabilities of a PHC-based EWS to enhance the existing dengue surveillance system in Brazil. To this end, we evaluated the performance of an early warning system based on Brazil's National Information System on PHC (SISAB) in terms of timeliness, sensitivity and positive predictive value (PPV) when compared to the existing epidemiological surveillance system. Data were collected from October 1, 2022, to March 1, 2024, and analyses were aggregated by epidemiological week and municipality.

### Local setting and data sources

Brazil has approximately 212.6 million people, living in 5570 municipalities.^16^ We analyzed data from the Brazilian Unified Health System (SUS), one of the largest public health systems globally, providing comprehensive healthcare to the entire population.^15^

Data for PHC encounters were extracted from SISAB, a database that registers data on all publicly funded PHC encounters in Brazil, coded by either the International Classification of Diseases (ICD-10) or the International Classification of Primary Care (ICPC-2). We compiled a list of ICD-10/ICPC-2 codes (refer to Supplementary Material) to extract weekly counts of arbovirus-related PHC encounters, per municipality. Since the diagnostic code for the reason of encounter at the PHC is usually based on symptoms and signs before laboratory results, we opted to maximize sensitivity by including codes for arbovirus diseases with dengue-like symptoms commonly occurring in Brazil (dengue, Zika, chikungunya, and, more recently, Oropouche fever).^17^

The existing Brazilian dengue surveillance system is reportedly one of the most comprehensive surveillance systems worldwide and has been largely used to describe the epidemiology of dengue.^18^^,^^19^ Reporting of all suspected dengue cases is mandatory in Brazil, and data is compiled in the National Information System on Notifiable Diseases (SINAN). A suspected dengue case is defined as acute fever (2–7 days) and two or more of the following: nausea or vomiting, rash, headache or retro-orbital pain, myalgia or arthralgia, petechiae or positive tourniquet test or leukopenia. A probable case is defined as any suspected case with a positive laboratory test (either serological or RT-PCR) or an epidemiological link to a confirmed case.^19^ The Ministry of Health (MoH) provides an openly available, non-identified SINAN database of all reported probable cases. We extracted data on probable dengue cases from SINAN on March 6th, 2025.

### Early warning and outbreak detection

The EWS was developed using the Early Aberration Reporting System (EARS), a widely used statistical method for detecting early signals of health events, such as outbreaks, in time series data.^20^^,^^21^ EARS is based on the cumulative sum (CUSUM) method, which computes a moving average over a baseline window to detect small changes in a time series.^20^ In this study, we employed two EARS variations: EARS-C1 and EARS-C2, which differ in how they select the weeks used to calculate the average, variance, and standard deviation. The mathematical formulation of both methods is detailed in the Supplementary Material.

The EARS is particularly suitable to operate with very short time series as a baseline.^21^ This feature provided a unique opportunity to our work, since the disruptions caused by the COVID-19 pandemic highly affected the stability of the PHC time series in Brazil.^22^ We applied the EARS-C1 and EARS-C2 methods to PHC time series to establish thresholds for early warning of arbovirus outbreaks.^23^ A warning is triggered when the observed number of arbovirus-related encounters in the week under analysis exceeds the quantile threshold (1−*α*) of the expected value. To evaluate the models, we used a moving window baseline of 8-week and a 12-week and examined three different quantile thresholds, corresponding to *α* values of 0.001, 0.05, and 0.10 to assess the best balance between sensitivity, PPV, and timeliness (see Supplementary Material).

We compared the PHC-based warnings to dengue outbreaks detected in the SINAN time series. To determine the starting point of outbreaks in the SINAN time series, we relied on the fixed-incidence thresholds of 100 per 100,000 inhabitants (lower risk) and 300 per 100,000 inhabitants (higher risk), as adopted by the Brazilian MoH.^24^ To calculate incidence, we considered the cumulative incidence in an 8-week rolling window. The end of the outbreak was established when the cumulative incidence in the rolling window fell below the fixed-incidence threshold. Additionally, we conducted the analysis stratified by population size of municipalities, categorized as small (up to 50,000 inhabitants), medium (50,000 to 100,000 inhabitants), or large (over 100,000 inhabitants).

### Performance evaluation

We evaluated the performance of the early warning system by assessing timeliness, sensitivity, and PPV.^25^^,^^26^

We defined timeliness as the time interval (in weeks) between a warning detected in the PHC time series and the fixed-incidence outbreak established in the SINAN time series. We restrained the evaluation of timeliness to PHC-based warnings associated with outbreaks. A PHC-based warning was considered associated with an outbreak if it was triggered in any week during the outbreak or within a window of 4 weeks of its occurrence. PHC-based warnings occurring outside this window were disregarded due to the uncertainty of being part of the same outbreak event.

Sensitivity was represented by the proportion of dengue outbreaks in the SINAN time series that were caught by the PHC-based warnings. It was estimated by dividing the number of dengue outbreaks caught by the PHC-based warnings by the total number of dengue outbreaks.^25^ The PPV was represented by the percentage of PHC-based warnings associated with dengue outbreaks. We estimated PPV by dividing the number of PHC-based warnings associated with dengue outbreak in the SINAN time series by the total number of PHC-based warnings.^25^ A high PPV indicates that most PHC-based warnings were issued when a SINAN outbreak was indeed occurring, thereby minimizing the issuance of false positives.^25^ Inconsistent warnings, i.e., warnings that were not supported by additional warnings for at least two consecutive weeks were regarded as false positives and included in the performance analysis as such.

All analyses were performed using R (version 4.0.5) and the surveillance package, version 1.20.0.^21^^,^^27^

### Research ethics

The study is based on secondary, aggregated, non-identified data, and was approved by the Ethical Review Board of Oswaldo Cruz Foundation–Instituto Gonçalo Moniz, Fiocruz Bahia, CAAE 61444122.0.0000.0040. All study procedures followed the ethical standards for research involving human beings, defined by resolution 466/2012 of the Brazilian National Health Council. The ethics committee granted a waiver for the need to obtain informed consent for data collection as the study was based on an aggregated database consisting of the number of encounters, per epidemiological week, per municipality, and per diagnostic code, with no information at the individual level.

### Role of the funding source

The funders did not interfere in the study design, data collection, analysis, interpretation or decision to submit the manuscript for publication.

## Results

We identified 2.8 million arbovirus-related PHC encounters (median = 43 encounters per week; Interquartile Range [IQR] = 11–199 encounters per week) and 2.7 million probable dengue reported cases (median = 37 encounters per week; IQR = 7–203 encounters per week) between October 1, 2022, and March 1, 2024. Both time series exhibited a markedly seasonal pattern, with numbers increasing in the initial epidemiological weeks of each year (Supplementary Figure S1-A). In the corresponding months in 2024, case numbers significantly exceeded those of previous years (Supplementary Figure S1-A). Arbovirus-related PHC encounters, and probable dengue reported cases were extensively distributed across Brazil: 5364 municipalities (96.3% of all 5570) recorded arbovirus-related PHC encounters, while 5269 (94.6%) reported dengue cases to SINAN (Supplementary Table S1). Only 75 (1.3%) municipalities had neither arbovirus-related PHC encounters nor probable dengue cases reported in the study period. All were small (≤50,000 inhabitants), with most (60 of 75) located in the subtropical South region (Supplementary Tables S1 and S2).

We observed little variation in performance metrics when comparing the EARS C1 and C2 variations, the 8-week and 12-week baselines, and the different *α*-values (Table 1 and Supplementary Table S4). Overall, the C1 variation showed higher sensitivity than the C2 variation, while the C2 variation demonstrated a higher PPV. The findings reported in the following text are based on EARS-C2 with a 12-week baseline and alpha value of 0.001, reflecting our priority to minimize false positive rates. The PPV ranged from 34.4% to 38.3% when using the 100/100,000 incidence threshold for outbreaks, with higher values registered in large municipalities. The sensitivity ranged from 75.0% in small municipalities to 90.0% in large municipalities when applying the 100/100,000 incidence threshold (Table 1).

**Table 1:** Distribution of performance estimates for the PHC-based early warning system using C1 and C2 variations of the Early Aberration Reporting System, across different baseline and alpha value settings.

Baseline	EARS-C1						EARS-C2					
	8-week (number of outbreaks = 5463)			12-week (number of outbreaks = 5432)			8-week (number of outbreaks = 5463)			12-week (number of outbreaks = 5391)		
**Alpha value (α)**	0.001	0.05	0.10	0.001	0.05	0.10	0.001	0.05	0.10	0.001	0.05	0.10
**Overall municipalities (n = 5570)**												
Sensitivity (%)	77.6	84.2	**85.5**	76.0	82.8	84.1	78.7	83.9	85.1	76.6	81.7	82.9
Timeliness (%)	56.6	65.9	**69.3**	57.3	65.7	67.8	58.1	66.0	68.6	57.2	65.2	67.2
PPV (%)	33.5	31.7	30.8	35.7	33.2	32.6	34.3	31.5	30.5	**36.5**	33.5	32.9
**Small municipalities (n = 4910)**												
Sensitivity (%)	76.5	83.0	**84.4**	74.7	81.6	82.9	77.2	82.6	83.9	75.0	80.4	81.7
Timeliness (%)	55.1	64.0	**67.4**	56.1	64.1	66.1	56.6	63.8	66.6	55.8	63.4	65.3
PPV (%)	33.6	32.2	31.5	35.6	33.7	33.2	34.5	32.1	31.2	**36.5**	33.9	33.4
**Medium municipalities (n = 334)**												
Sensitivity (%)	82.8	92.4	**93.3**	82.0	91.2	91.8	86.9	92.3	93.0	85.9	90.0	90.3
Timeliness (%)	64.1	78.3	**79.8**	62.6	74.6	76.7	67.2	79.5	81.2	66.1	76.0	78.4
PPV (%)	30.4	26.1	24.5	33.9	28.8	27.4	30.9	25.7	24.0	**34.4**	29.1	28.1
**Large municipalities (n = 316)**												
Sensitivity (%)	87.2	92.6	93.8	88.3	91.8	93.1	91.7	93.9	**94.4**	90.0	92.7	93.4
Timeliness (%)	69.6	79.2	**84.2**	67.7	79.0	80.9	70.0	81.6	83.0	67.8	77.5	81.1
PPV (%)	34.8	30.1	28.3	38.3	31.6	29.6	35.1	29.4	27.5	**38.3**	32.3	30.7

Performance was evaluated based on outbreaks detected in the SINAN time series using a 100/100,000 fixed-incidence threshold.

Small municipalities: ≤50,000 inhabitants; Medium municipalities: 50,000–100,000 inhabitants; Large municipalities: ≥100,000 inhabitants. Timeliness refers to a warning being triggered from four weeks prior to the same week the outbreak is detected, and PPV refers to the percentage of warnings associated with outbreaks. The bold values in the table indicate the best performance for each metric.

EARS, Early Aberration Reporting System. PHC, Primary Health Care. PPV, Positive Predictive Value. SINAN, National Information System on Notifiable Diseases.

**Fig. 1:**
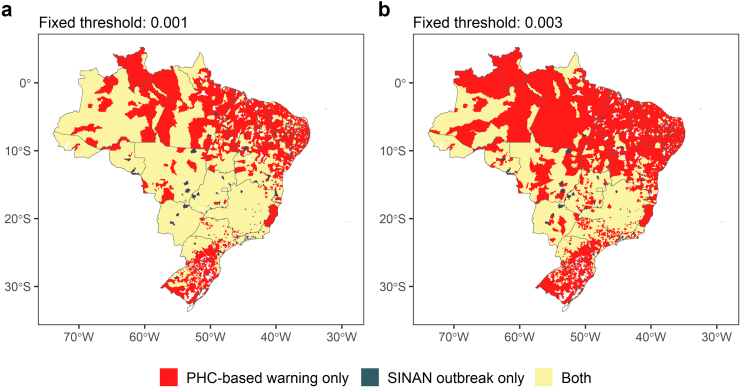
**Distribution of PHC-based warnings (EARS-C2 with a 12-week baseline and an alpha value of 0.001) and SINAN outbreak detection based on fixed thresholds of: a) 100 cases per 100,000 inhabitants; and b) 300 cases per 100,000 inhabitants**. The color red indicates municipalities that presented PHC-based warnings only (a) n = 2076; b) n = 2956); charcoal blue indicates municipalities with SINAN outbreaks only (a) n = 126; b) n = 63); yellow represents municipalities presenting both (a) n = 3057; b) n = 2177); and white represents municipalities without PHC-based warnings or detected SINAN outbreaks (a) n = 311; b) n = 374). EARS, Early Aberration Reporting System. PHC, Primary Health Care. SINAN, National Information System on Notifiable Diseases.

PHC-based warnings were more broadly distributed geographically when compared to outbreak detection using the SINAN time series (Fig. 1). In 5133 (92.2%) municipalities, at least one PHC-based warning was detected, whereas an outbreak based on the SINAN time series was detected in 3183 (57.1%) municipalities (Supplementary Table S3). Conversely, 374 (6.7%) municipalities, all with a population size of up to 50,000 inhabitants and mainly located in the South and Northeast regions, presented no PHC-based warnings or SINAN outbreaks (Fig. 1).

**Fig. 2:**
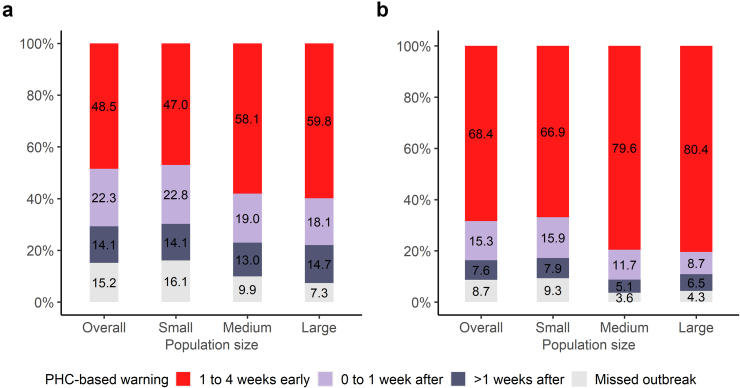
Distribution of PHC-based warnings (EARS-C2 with a 12-week baseline and an alpha value of 0.001) associated with SINAN outbreaks according to the time interval between the warning and the outbreak detection, and municipality population size. Outbreak detection in the SINAN time series was based on: a) a fixed-incidence threshold of 100/100,000 (n = 2361 municipalities); and b) a fixed-incidence threshold of 300/100,000 (n = 1583 municipalities). Small municipalities: ≤50,000 inhabitants; Medium municipalities: 50,000 to 100,000 inhabitants; Large municipalities: ≥100,000 inhabitants. EARS, Early Aberration Reporting System. PHC, Primary Health Care. SINAN, National Information System on Notifiable Diseases.

When assessing the timeliness of the PHC-based warning system, we found that consistent warnings anticipated 48.5%, and 68.4% of outbreaks identified in the SINAN time series (Fig. 2). Overall, the proportion of timely warnings was higher among medium and larger municipalities (Fig. 2). When using the incidence of 100 cases per 100,000 inhabitants, PHC-based warnings anticipated approximately 60.0% of SINAN outbreaks in large municipalities (over 100,000 inhabitants) (Fig. 2A), and when using the incidence of 300/100,000 inhabitants 80.0% of SINAN outbreaks could be anticipated (Fig. 2B). Supplementary Figures S1-B, C, and D show results of the EWS for representative municipalities of small, medium, and large sizes.

## Discussion

This study used PHC administrative data to develop an EWS for dengue outbreaks in Brazil. We found that the PHC-based EWS successfully identified between 76.0% and 85.5% of dengue outbreaks detected by the SINAN-based surveillance system currently in practice. Notably, it detected up to 69.3% of outbreaks up to four weeks earlier. Early detection was particularly effective in larger municipalities, with dengue incidence timeliness of approximately 70.0% for a fixed incidence threshold of 100 per 100,000 inhabitants, and 90.0% for 300 cases per 100,000. These results underscore the critical role of PHC administrative data in strengthening complementary surveillance systems and enabling a timely outbreak response.

Importantly, the PHC-based EWS provided broader coverage of epidemiological surveillance than systems relying solely on active case notification. Our analysis revealed that 95 additional municipalities recorded arbovirus-related PHC encounters than those reporting dengue cases to SINAN, with warnings being triggered across a wider range of geographical areas particularly in the South, Northeast and North regions. This wider reach not only highlights the potential of PHC-based data in capturing early warning signals but also underscores its ability to complement existing surveillance systems, particularly in regions with limited active case notification. Furthermore, the mandatory nature of administrative data—such as registering health services for accountability—ensures the maintenance of a high-quality, updated database.^28^ Thus, EWS based on administrative data offers substantial advantages over those relying exclusively on case notifications.

Passive surveillance data have proven effective in detecting long-term trends,^19^ but capturing the magnitude of an epidemic within a short period remains a challenge due to delays in notification schedules, which can undermine the effectiveness of the EWS.^8^^,^^29^ Correction techniques to adjust for these delays are commonly employed in passive surveillance data.^29^ In Brazil for these techniques, when applied within a flexible statistical modeling framework, enable nowcasting of the observed data.^29^^,^^30^ Additionally, participatory surveillance based on mobile devices was tested in the country as an option to booster passive surveillance results.^31^ Underreporting is another recognized challenge in passive surveillance systems, with estimates ranging from 9 to 21 actual cases for every reported case.^7^^,^^12^ In our study, there were more cities reporting arbovirus-related PHC encounters than dengue cases to the surveillance system. A small fraction of cities reported neither, with most of them (80%) concentrated in the subtropical South region, where endemic dengue virus circulation has only recently been observed.^2^ Underreporting may be associated with limitations in human resources and infrastructure, such as low computational capacity and unstable internet connections.^12^ Our study presents an alternative approach by utilizing PHC administrative data, which is routinely collected on a national scale, offering a more immediate and robust solution for outbreak detection.

Our findings align with those of Ledien et al.,^32^ who tested different EWS approaches in Cambodia using case reports, including the EARS algorithm. Their study highlighted the effectiveness of a Bayesian approach, though it did not assess the timeliness of outbreak detection. The authors emphasized that the ease of interpreting results and cost-effectiveness stand out as essential features for the adoption of these systems by decision-makers.^32^ Similarly, a systematic review on modeling tools for dengue risk mapping concluded that, although advancing on complex modeling methodology improved data analysis and visualization, the majority of studies were based on retrospective data and the limited availability of resources and data quality affects the applicability of these tools for EWS in real settings.^10^ In this context, the EARS algorithm proves particularly valuable, as it requires minimal data—only three time points for analysis—and relies solely on the event under surveillance as an independent variable. In our study, its application to routinely collected PHC administrative data enhances its utility, offering a scalable and easily interpretable solution for outbreak detection.

In our study, smaller municipalities showed lower performance than medium- and large-sized municipalities. Since dengue outbreaks exhibit spatial and temporal correlation and are heavily influenced by human mobility,^33^ outbreak situations in smaller settings could be informed by EWS results from the nearest medium or large size municipality, or from its immediate geographic region. These regions comprise groups of nearby municipalities forming an urban network based on dependency relationships and population movement for goods and services.^34^ Leveraging regional signals offers a simple, effective approach to overcome the challenges in implementing the EWS in smaller municipalities.

The inclusion of climate, meteorological and entomological variables for the development of EWS is commonly reported in the literature.^8^^,^^35^ However, regular access to consistent and updated meteorological data with spatial scale compatible with EWS analysis is a challenging issue in many settings, particularly in low- and middle-income countries (LMIC),^9^^,^^36^^,^^37^ and entomological surveillance data is often scarce.^35^ This limits the use of these EWS integrated into routine surveillance.^10^ For instance, an EWS in Mexico using active case notification and climatological data achieved higher standards than the numbers presented here (100% sensibility, and 83% positive predictive value). However, the performance evaluation was limited to 9 out of 137 endemic regions, because the necessary complete set of variables was only available for those regions.^36^

Two recent reviews on EWS for dengue outbreaks found that most studies included multiple variables—such as climate, meteorological and entomological variables—integrated into the EWS.^8^^,^^35^ Hussain-Alkhateeb et al.^8^ found 28 studies, with 24 of them using at least one meteorological variable. The studies reported a range of sensitivity between 40% and 100%, an interval that includes the results we presented here. However, only five of the studies reviewed showed the feasibility of being integrated into existing surveillance systems, and almost all presented tools requiring highly skilled users. The authors concluded that most EWS remain in the academic field, and there is a need for more pragmatic and context-adapted EWS tools to support the global health agenda for outbreak control.^8^ Baharom et al. retrieved 17 studies, 15 of them including meteorological variables into the EWS. Similarly, the reported sensitivity ranges from 50% to 100%, an interval that includes the results we show here.^35^ The authors did not report on the implementation of the EWS in real-case scenarios.

In our study, we tested EARS C1 and C2 variations with different parameterizations and baseline periods of 8 and 12 weeks. All analyses yielded similar results, with minimal variation in performance metrics. These findings represent the average across municipalities, and the best-performing configuration overall may not be optimal for each municipality. Therefore, given our goal of proposing a practical, easy-to-implement EWS algorithm, we prioritized usability over identifying a universal best model. Furthermore, it is important to highlight that the optimum trade-off between sensitivity, PPV and timeliness depends more on the public health strategy adopted by authorities than on statistical measures.

The EARS algorithm is especially suited for situations with limited historical data, requiring as few as three time points for threshold setting.^23^ However, this flexibility comes at the cost of controlling for seasonality, which can lead to alarms being triggered during seasonal peaks. Methods that adjust for seasonality tend to achieve higher PPV, although usually depending on longer, stable time series.^38^ Among them, the widely used improved Farrington algorithm.^26^ In our scenario, disruptions caused by the COVID-19 pandemic significantly affected the stability of the PHC time series, hindering the feasibility of methods requiring longer baseline data.^22^ Our findings show an overall PPV ranging from 30.5% to 36.5%, implying a considerable proportion of false alarms. These results align with the work by Nekorchuk et al.,^25^ who reported a moderate early detection rate (43%–87%) and a low true positive rate (25%–40%) when applying EARS variations to real malaria surveillance data.^25^ Their study found the improved Farrington algorithm to be most effective, as it achieved the best trade-off on maximizing both sensitivity (>70%) and specificity (>70%). Bédubourg & Le Strat,^38^ compared 21 early warning methods using simulated data, concluding that no single method achieved outbreak detection performance sufficient for reliable large-scale surveillance.^38^ In line with this, in this study we present the feasibility of establishing a PHC-based EWS as a complementary system to enhance dengue surveillance.

Dengue and other emerging arboviruses represent a major public health issue, and increasing EWS capacities is crucial for timely clinical preparations and vector control,^12^ reducing the negative impacts of the disease on vulnerable populations,^4^ and the economy.^1^ In this context, the feasibility of technology transfer and cost-effectiveness should be considered as necessary features of novel surveillance tools. With the global rise of dengue cases each year, and the environmental suitability for dengue transmission and risk of importation reaching different continents,^39^, ^40^, ^41^ predictive modeling research should address the perspective of implementing EWS within public health surveillance programs. In this context, syndromic surveillance at the PHC level emerges as an inclusive and scalable approach, particularly in resource-limited regions. Using routinely collected administrative data, such as PHC data, avoids the need for dedicated personnel or infrastructure. This is notably beneficial in Brazil, with its universal healthcare system, which provides granular coverage across all country, and its nationwide information systems.^42^

Additionally, the EWS presented in this study is part of the broader AESOP initiative,^13^ which relies on open source tools codes to provide for a feasible and readily accomplished embedding process within the surveillance routine. This approach ensured a successful collaboration between the AESOP initiative and the MoH surveillance team, which ultimately led to the inclusion of the PHC-based EWS in the MoH's National Plan for Reducing Dengue and Other Arboviruses.^43^

Our study has some limitations. First, the PHC database includes only encounters from publicly funded healthcare facilities, which account for approximately 75% of health assistance in Brazil.^42^ Outbreaks emerging among the wealthier stratum of the population may reduce the timeliness and sensitivity of the EWS. Additionally, we acknowledge that dengue cases remain underrepresented, as a substantial proportion of infections are asymptomatic or present with mild, flu-like symptoms, often resulting in patients not seeking health care.^44^^,^^45^ Therefore, underreporting persists, and may affect the EWS performance. Second, the system's performance was lower in smaller municipalities—likely due to limited health and technological infrastructure. Addressing these regional disparities to strengthen surveillance is critical to ensuring equitable detection capacity and control of dengue epidemics.

### Conclusions

This study demonstrates the feasibility of enhancing dengue surveillance in Brazil by integrating PHC administrative data into an EWS. The PHC-based EWS successfully identified most outbreaks detected by the existing surveillance system while providing warnings up to four weeks in advance. Its reliance on routinely collected data enables broader geographic coverage, reaching regions with limited human resources and infrastructure. Additionally, its automated feature and reliability on open source tools and codes enhances scalability, which is crucial for preparedness and response to health emergencies, given that data collection during an early outbreak phase is time-consuming. Integrating the PHC-based EWS approach to complement the established passive notification system will likely leverage the strengths of both approaches, improving early detection and accuracy. Furthermore, our findings align with global health priorities of improving epidemic detection using digital surveillance through multidisciplinary data integration for effective deployment of response measures.

## Contributors

RSS, PTVF, IM, and MB-N conceived the idea of the study; RSS and PTVF conducted the analysis and JB, GCGB, and TCS verified the scripts. All authors contributed to the study design. VdAO was responsible for data obtaining, VdAO, JB, and GCGB were responsible for data curating and processing; VdAO, JB, and GCGB accessed and verified the data. RSS, PTVF, and IM wrote the first draft, RSS, MB-N, TCS, PIPR, IM, VSB, and GOP further revised the manuscript. All authors critically revised the manuscript and approved the final version for submission.

## Data sharing statement

Our agreement with the Brazilian MoH for accessing the referenced databases patently denies authorization of access to any third parties. All requests to access these databases must be addressed to the Brazilian MoH. The corresponding code to ensure reproducibility of our results is available in a GitHub repository [https://github.com/cidacslab/AESOP-Data-Documentation/tree/main/DataPipeline/manuscript%20arboviroses].

## Editor's note

The Lancet Group takes a neutral position with respect to territorial claims in published maps and institutional affiliations.

## Declaration of interests

All the authors declared no competing interests.

## Appendix A Supplementary data

Translated-summary
