# Supplementary material for: Primary health care data-based early warning system for dengue outbreaks: a nationwide case study in Brazil
Source: Lancet Reg Health Am. 2025 Jul 4;48:101165. doi: 10.1016/j.lana.2025.101165 (PMC12270639; doi:10.1016/j.lana.2025.101165)
Supplement: Translated-summary [file mmc1.docx]

**Sistema de alerta precoce baseado em dados da atenção primária à saúde para surtos de dengue: um estudo de caso nacional no Brasil.**

Rejane Santos-Silva^1,†^, Pilar Tavares Veras Florentino^1,2,†^, Thiago Cerqueira-Silva^2,3^, Vinicius de Araújo Oliveira^1,4^, Juracy Bertoldo Junior^1^, George C. G. Barbosa^1^, Gerson O. Penna^5,6^, Viviane S. Boaventura^2,4^, Pablo I. Pereira Ramos^1^, Manoel Barral-Netto^1,2,4,‡^, Izabel Marcilio^1,7,‡ ,^*

^1^ Centro de Integração de Dados e Conhecimentos para Saúde (Cidacs), Instituto Gonçalo Moniz, Fundação Oswaldo Cruz (Fiocruz Bahia), Salvador, Brazil

^2^ Laboratório de Medicina e Saúde Pública de Precisão, Fundação Oswaldo Cruz, Salvador, Brazil

^3^ Faculty of Epidemiology and Population Health, London School of Hygiene and Tropical Medicine, London, United Kingdom

^4^ Faculdade de Medicina da Bahia, Universidade Federal da Bahia, Salvador, Brazil

^5^ Núcleo de Medicina Tropical, Universidade de Brasília, Brasília, Brazil

^6^ Escola de Governo Fiocruz Brasília, Fiocruz Brasília, Brasília, Brazil

^7^ Escola Bahiana de Medicina e Saúde Pública, Salvador, Brazil

^†^Esses autores contribuíram igualmente neste artigo

^‡^co-autores seniors.

^∗^Corresponding author. I. Marcilio Centro de Integração de Dados e Conhecimento para Saúde, Instituto Gonçalo Moniz, Fundação Oswaldo Cruz, Parque Tecnológico Edf. Tecnocentro, Rua Mundo 121, sala 315, Salvador, BA 41745-715, Brazil.

**Resumo**

**Introdução:** A vigilância epidemiológica tradicional apresenta limitações para a detecção precoce de surtos e epidemias. Dados administrativos da Atenção Primária à Saúde (APS) aplicados à vigilância sindrômica representam uma solução custo-efetiva para apoiar sistemas de alerta precoce (SAPs). Este estudo avaliou a performance de um SAP para epidemias de dengue utilizando dados da APS no Brasil.

**Métodos:** Para estabelecer o SAP, o Sistema de Notificação Precoce de Anomalias (EARS-C1 e EARS-C2) foi aplicado à série temporal de atendimentos por arboviroses na APS, entre 1º de outubro de 2022 e 1º de março de 2024, nos 5.570 municípios do país. A performance do sistema foi avaliada pela estimativa da tempestividade, sensibilidade e valor preditivo positivo (VPP) em comparação à detecção de surtos baseada na vigilância epidemiológica tradicional.

**Resultados:** Foram registrados atendimentos por arboviroses na APS e notificações de dengue em 5.364 (96,3%) e 5.269 (94,6%) municípios brasileiros, respectivamente. Os alertas baseados na APS anteciparam 48,5% (100 casos/100.000 habitantes) e 68,4% (300/100.000) dos surtos detectados pela vigilância epidemiológica tradicional. A tempestividade foi maior em municípios com mais de 100.000 habitantes.

**Interpretação:** O algoritmo EARS aplicado aos dados da APS antecipou surtos em até quatro semanas à detecção a partir da vigilância tradicional, e o uso de dados administrativos coletados na rotina dos serviços assegura maior cobertura e escalabilidade do sistema. Este estudo demonstrou a viabilidade de se integrar dados administrativos da APS em um SAP para detecção precoce de epidemias de dengue no Brasil.

**Financiamento:** Iniciativa de Saúde da Fundação Rockefeller e Fundação de Amparo à Pesquisa do Estado da Bahia (FAPESB), Brasil.

**Palavras-chave:** sistema de alerta precoce, dengue, arbovírus, vigilância
