# Supplementary material for: Primary health care data-based early warning system for dengue outbreaks: a nationwide case study in Brazil
Source: Lancet Reg Health Am. 2025 Jul 4;48:101165. doi: 10.1016/j.lana.2025.101165 (PMC12270639; doi:10.1016/j.lana.2025.101165)
Supplement: Supplementary Material [file mmc2.docx]

**Title:** *Primary health care data-based early warning system for dengue outbreaks: A nationwide case study in Brazil*

**Journal name:** The Lancet Regional Health - Americas

**Author information:**

Rejane Santos-Silva^1,†^, Pilar Tavares Veras Florentino^1,2,†^, Thiago Cerqueira-Silva^2,3^, Vinicius de Araújo Oliveira^1,4^, Juracy Bertoldo Junior^1^, George C. G. Barbosa^1^, Gerson O. Penna^5,6^, Viviane S. Boaventura^2,4^, Pablo I. Pereira Ramos^1^, Manoel Barral-Netto^1,2,4,‡^, Izabel Marcilio^1,7,‡ ,^*

^1^ Centro de Integração de Dados e Conhecimentos para Saúde (Cidacs), Instituto Gonçalo Moniz, Fundação Oswaldo Cruz (Fiocruz Bahia), Salvador, Brazil

^2^ Laboratório de Medicina e Saúde Pública de Precisão, Fundação Oswaldo Cruz, Salvador, Brazil

^3^ Faculty of Epidemiology and Population Health, London School of Hygiene and Tropical Medicine, London, United Kingdom

^4^ Faculdade de Medicina da Bahia, Universidade Federal da Bahia, Salvador, Brazil

^5^ Núcleo de Medicina Tropical, Universidade de Brasília, Brasília, Brazil

^6^ Escola de Governo Fiocruz Brasília, Fiocruz Brasília, Brasília, Brazil

^7^ Escola Bahiana de Medicina e Saúde Pública, Salvador, Brazil

^†^These authors contributed equally to this work

^‡^ co-senior authors

*Correspondence: I. Marcilio Centro de Integração de Dados e Conhecimento para Saúde, Instituto Gonçalo Moniz, Fundação Oswaldo Cruz. Parque Tecnológico Edf. Tecnocentro, Rua Mundo 121, sala 315, Salvador, BA 41745-715, Brasil. izabel.souza@fiocruz.br

# *Supplementary materials*

**ICD-10 and ICPC-2 codes for Primary Health Care (PHC) encounters**

Codes for PHC encounters extracted from SISAB, classified using the International Classification of Diseases (ICD-10) and the International Classification of Primary Care (ICPC-2). The full list is available in the GitHub repository: [AESOP Data Documentation](https://github.com/cidacslab/AESOP-Data-Documentation/blob/main/DataPipeline/documentation/code_list_arbovirus_apr2024.csv).

| **Type** | **Code** | **Name** |
| --- | --- | --- |
| CIAP | A77 | Dengue and other viral diseases |
| CID 10 | A90 | Dengue |
| CID 10 | A91 | Dengue hemorrhagic fever |
| ABP | ABP019 | Severe dengue |
| CID | A972 | Dengue grave |
| CID | A979 | Unspecified dengue |
| CID 10 | A920 | Chikungunya |
| CID 10 | A92 | Other mosquito-borne viral fevers |
| CID 10 | A928 | Other specified mosquito-borne viral fevers |
| CID 10 | A929 | Mosquito-borne viral fever, unspecified |
| CID 10 | A99 | Unspecified viral hemorrhagic fevers |
| CID 10 | A98 | Other viral hemorrhagic fevers |
| CID | A925 | Zika |
| CID | A93 | Other arboviral diseases |
| CID | A988 | Other viral hemorrhagic fevers |
| CID | A93 | Oropouche fever |

**Early Aberration Reporting System (EARS)**

We briefly describe the *Early Aberration Reporting System* (EARS) algorithm in its C1 and C2 variations used in this study. EARS is a widely used method for detecting early signals of health events, such as outbreaks, using time series data.^1,2^ This system has two variations that are employed in our study: EARS-C1 and EARS-C2. Both methods are based on CUSUM (Cumulative Sum) principles and compute a moving average using a baseline window (B).^2,3^ However, they differ in how they select the weeks used to calculate the average ($\underline{Y}(t)$), variance ($S^{2}\left( t \right)$), and standard deviation ($S\left( t \right)$). In the EARS-C1 method, these calculations consider the B preceding weeks (from *t* − B to *t* − 1).^1^ In contrast, in the EARS-C2 method, the three most recent weeks are excluded, and the mean, variance, and standard deviation are computed based on the weeks from *t* − B to *t* − 3.^1^ As described in the *Method* section, we used B = 8 and B = 12.

Under the null hypothesis of no outbreak (C(*t*) ~ *N*(0,1)), a warning is triggered when C(*t*) ≥ z-α, where z-α represents the (1 – α) quantile of the standard normal distribution, assuming that the weekly counts within the moving window follow this distribution. In this study, we used α = 0.001, 0.05, and 0.01. The mathematical details and equations underlying the EARS-C1 and EARS-C2 methods are available below:

|  | **EARS-C1** | **EARS-C2** |
| --- | --- | --- |
| *Average* | $\underline{Y}₁(t)=\frac{1}{B}\sum_{i=t-B}^{t-1} Y(i)$ | $\underline{Y}_{2}\left( t \right)=\frac{1}{B}\sum_{i=t-3}^{t-(B-2)} Y\left( i \right)$ |
| *Variance* | $S_{1}^{2}\left( t \right)=\frac{1}{B}\sum_{i=t-1}^{t-B} \left[ Y\left( i \right)- \underline{Y}_{1}\left( i \right) \right]^{2}$ | $S_{2}^{2}\left( t \right)=\frac{1}{B-1}\sum_{i=t-3}^{t-(B-2)} \left[ Y\left( i \right)- \underline{Y}_{2}\left( i \right) \right]^{2}$ |
| *Standard deviation* | $S_{1}\left( t \right)= \sqrt{S_{1}^{2}}(t)$ | $S_{2}\left( t \right)= \sqrt{S_{2}^{2}}(t)$ |
| *z-score* | $C₁(t)=\frac{Y_{1}\left( t \right)-\underline{Y}_{1}\left( t \right)}{S_{1}(t)}$ | $C_{2}(t)=\frac{Y_{2}\left( t \right)-\underline{Y}_{2}\left( t \right)}{S_{2}(t)}$ |
| *Upperbound* | $U_{1}\left( t \right)=\underline{Y}_{1}\left( t \right)+[z_{1}- \alpha]\times S_{1}(t)$ | $U_{2}\left( t \right)=\underline{Y}_{2}\left( t \right)+{[z}_{2}- \alpha]\times S_{2}(t)$ |


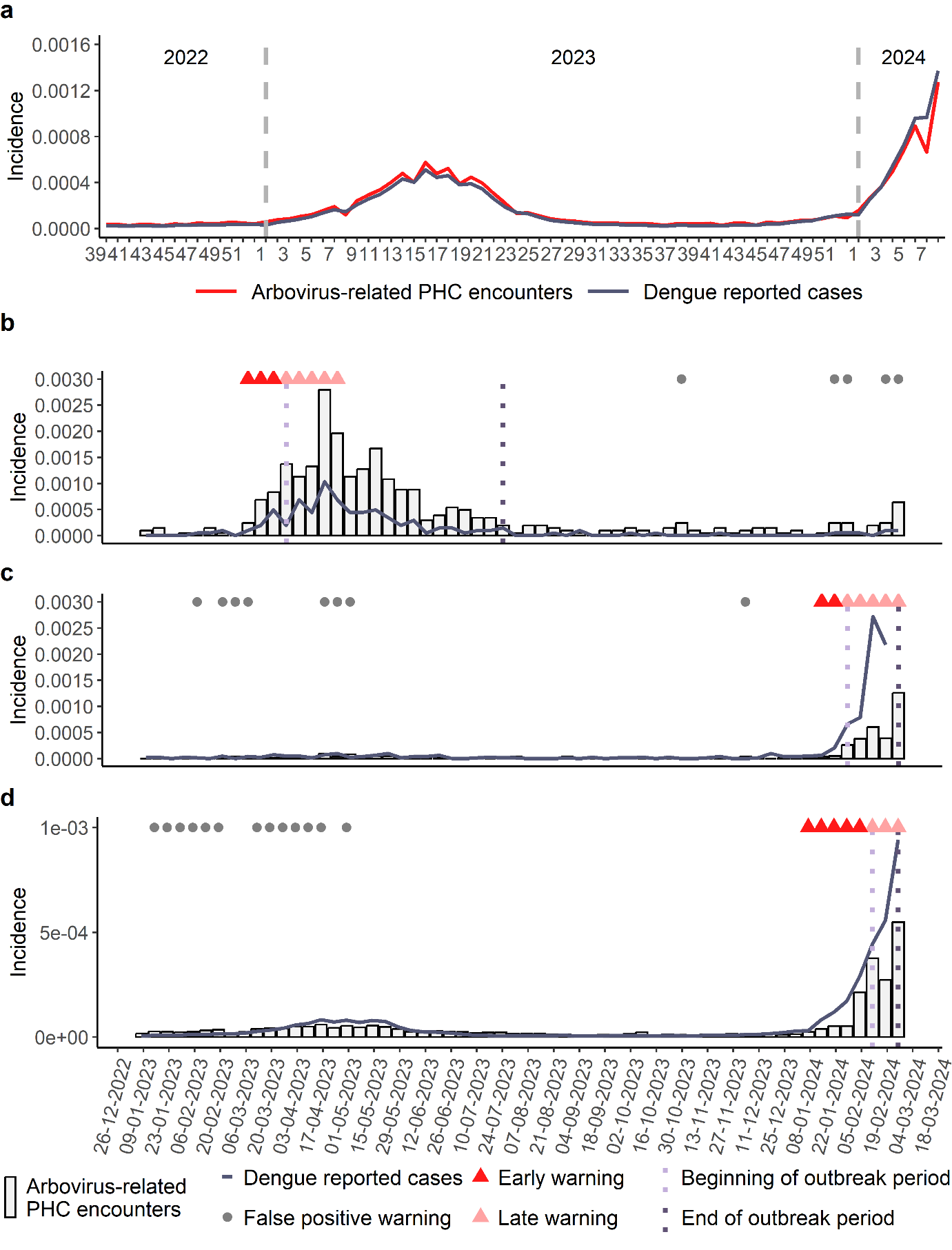


**Fig. S1 Distribution of incidence (number of records divided by the population) of arbovirus-related PHC encounters and dengue reported cases in Brazil from October 2022 to March 2024.** a) Incidence of arbovirus-related PHC encounters and dengue-reported cases across 74 epidemiological weeks in Brazil. Early outbreak signals detected by EARS warnings (C2 variation with a 12-weeks baseline and alpha 0.001) in b) Fátima do Sul, Mato Grosso do Sul state (≤ 50,000 inhabitants); c) Lorena, São Paulo state (from 50,000 to 100,000 inhabitants); and d) São Paulo, São Paulo state (≥ 100,000 inhabitants). The gray bars represent the incidence (number of encounters divided by the population) of arbovirus-related PHC encounters, and the charcoal blue curve shows the incidence of probable dengue reported cases. The dashed vertical lines indicate the start and end of the identified outbreak. red triangles mark warnings triggered before the first outbreak signal, while light red triangles indicate late warnings. Gray circles represent warnings triggered without a corresponding outbreak event. EARS = Early Aberration Reporting System. PHC = Primary Health Care.


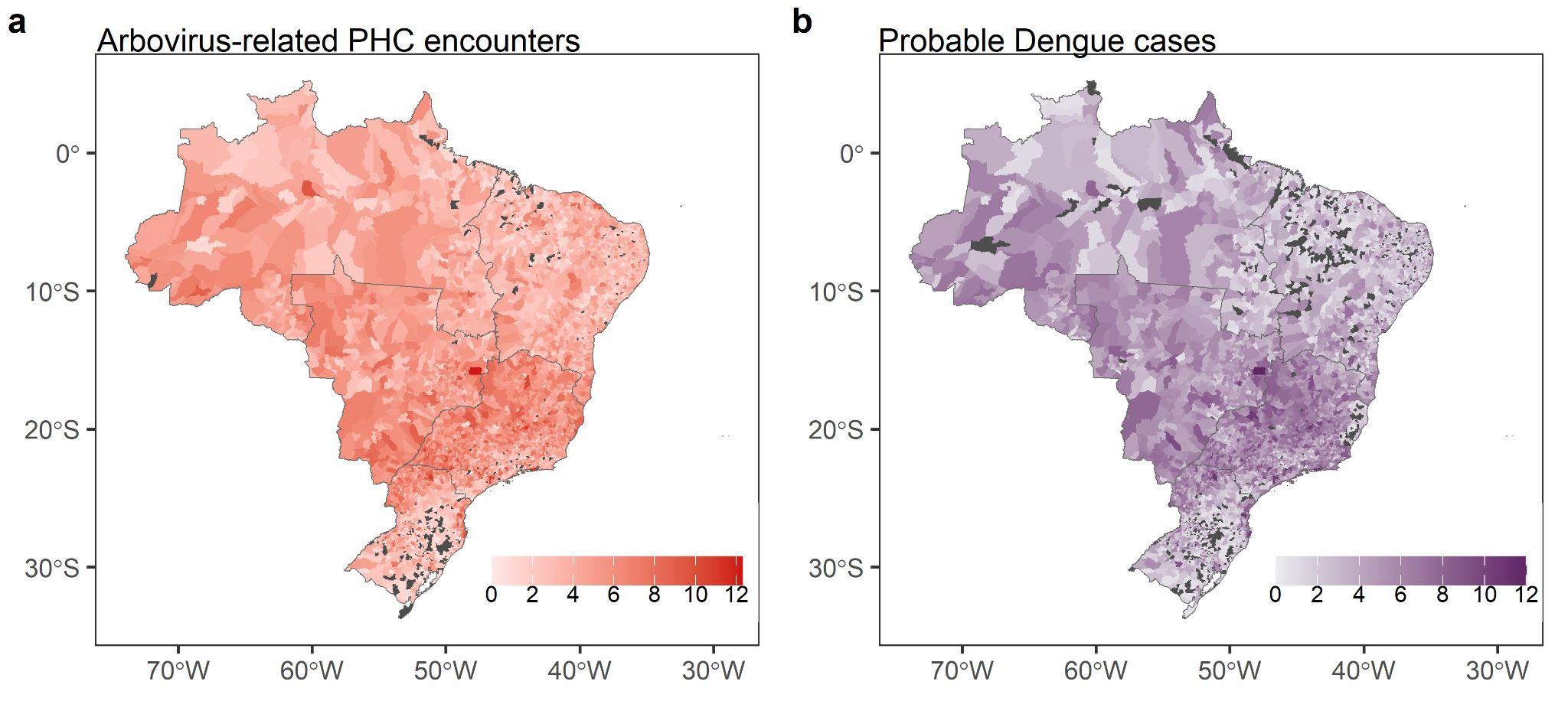


**Fig. S2 Distribution of arbovirus-related PHC encounters and probable dengue cases reported to SINAN.** a) Arbovirus-related PHC encounters; b) Probable dengue cases reported to SINAN. Values were log-transformed (log + 1). Municipalities with zero records during the evaluated period are depicted in dark gray. PHC = Primary Health Care. SINAN = National Information System on Notifiable Diseases.

**Table S1 Distribution of arbovirus-related PHC encounters and probable dengue report to SINAN according to municipalities population size (Small ≤ 50,000 inhabitants; Medium from 50,000 to 100,000 inhabitants; and Large ≥ 100,000 inhabitants).** PHC=Primary Health Care. SINAN=National Information System on Notifiable Diseases.

| **Population size** | **Municipalities (n)** | **Arbovirus-related PHC encounters reported n [%]** | **Probable dengue cases reported n [%]** | **No arbovirus-related PHC encounter or probable dengue cases report n [%]** |
| --- | --- | --- | --- | --- |
| Brazil | 5570 | 5364 [96.3%] | 5269 [94.6%] | 75 [1.3%] |
| Small | 4910 | 4705 [95.8%] | 4609 [93.9%] | 75 [1.5%] |
| Medium | 344 | 343 [99.7%] | 344 [100.0%] | 0 [0.0%] |
| Large | 316 | 316 [100.0%] | 316 [100.0%] | 0 [0.0%] |

**Table S2 Distribution of arbovirus-related PHC encounters and probable dengue report to SINAN according to geographical region.** PHC = Primary Health Care. SINAN = National Information System on Notifiable Diseases.

| **Geographical region** | **Municipalities (n)** | **Arbovirus-related PHC encounters reported n [%]** | **Probable dengue cases reported n [%]** | **No arbovirus-related PHC encounter or probable dengue cases report n [%]** |
| --- | --- | --- | --- | --- |
| Brazil | 5570 | 5364 [96.3%] | 5269 [94.6%] | 75 [1.3%] |
| North | 450 | 441 [98.0%] | 436 [96.9%] | 1 [0.2%] |
| Northeast | 1794 | 1748 [97.4%] | 1676 [93.4%] | 12 [0.7%] |
| Central-West | 467 | 466 [99.8%] | 466 [99.8%] | 0 [0.0%] |
| Southeast | 1668 | 1646 [98.7%] | 1634 [98.0%] | 2 [0.1%] |
| South | 1191 | 1063 [89.3%] | 1057 [88.7%] | 60 [5.0%] |

**Table S3 - Distribution of PHC-based warnings (EARS-C2 with a 12-week baseline and an alpha value of 0.001) and outbreaks detected using the 100/100,000 inhabitants and 300/300,000 inhabitants fixed-incidence according to municipalities population size (Small ≤ 50,000 inhabitants; Medium from 50,000 to 100,000 inhabitants; and Large ≥ 100,000 inhabitants).** PHC = Primary Health Care. EARS = Early Aberration Reporting System

| **Population size** | **Municipalities (n)** | **PHC-based warnings n [%]** | **Outbreaks detected - Incidence threshold of 0.001 n [%]** | **Outbreaks detected - Incidence threshold of 0.003 n [%]** |
| --- | --- | --- | --- | --- |
| Brazil | 5,570 | 5,133 [92.2] | 3,183 [57.1] | 2,240 [40.2] |
| Small | 4,910 | 4,481 [91.3] | 2,792 [56.9] | 1,979 [40.3] |
| Medium | 344 | 377 [98.0] | 195 [56.7] | 132 [38.4] |
| Large | 316 | 315 [99.7] | 196 [62.0] | 129 [40.8] |

**Table S4. Distribution of performance estimates for the PHC-based early warning system using C1 and C2 variations of the Early Aberration Reporting System, across different baseline and alpha value settings. Performance was evaluated based on outbreaks detected in the SINAN time series using a 300/100,000 fixed-incidence threshold.** Small municipalities: ≤ 50,000 inhabitants; Medium municipalities: 50,000 to 100,000 inhabitants; Large municipalities: ≥ 100,000 inhabitants. Timeliness refers to a warning being triggered from four weeks prior to the same week the outbreak is detected, and PPV refers to the percentage of warnings associated with outbreaks. Bold values indicate the best performance for each metric. EARS = Early Aberration Reporting System. PHC = Primary Health Care. PPV = Positive Predictive Value. SINAN = National Information System on Notifiable Diseases.

|  | **EARS-C1** | | | | | | **EARS-C2** | | | | | |
| --- | --- | --- | --- | --- | --- | --- | --- | --- | --- | --- | --- | --- |
| **Baseline** | **8-week**  **(number of outbreaks = 3,227)** | | | **12-week**  **(number of outbreaks = 3,219)** | | | **8-week**  **(number of outbreaks = 3,227)** | | | **12-week**  **(number of outbreaks = 3,227)** | | |
| **Alpha value (α)** | 0.001 | 0.05 | 0.10 | 0.001 | 0.05 | 0.10 | 0.001 | 0.05 | 0.10 | 0.001 | 0.05 | 0.10 |
| ***Overall municipalities (n = 5,570)*** |  |  |  |  |  |  |  |  |  |  |  |  |
| Sensitivity (%) | 81.2 | 87.3 | **89.0** | 79.7 | 86.5 | 87.9 | 83.6 | 87.7 | 88.9 | 81.6 | 85.7 | 86.7 |
| Timeliness (%) | 72.5 | 81.0 | **83.0** | 73.5 | 80.5 | 81.8 | 76.4 | 81.3 | 83.0 | 76.2 | 81.0 | 81.8 |
| PPV (%) | 20.1 | 18.5 | 17.7 | 21.5 | 19.5 | 18.9 | 20.8 | 18.3 | 17.5 | **22.3** | 19.7 | 19.2 |
| ***Small municipalities (n = 4,910)*** |  |  |  |  |  |  |  |  |  |  |  |  |
| Sensitivity (%) | 80.7 | 86.6 | 88.3 | 79.1 | 85.7 | 87.1 | 82.8 | 86.9 | 88.2 | 80.7 | 84.8 | 81.7 |
| Timeliness (%) | 71.2 | 79.4 | 81.7 | 72.2 | 78.9 | 80.3 | 74.8 | 79.9 | 81.6 | 74.7 | 79.5 | 65.3 |
| PPV (%) | 20.3 | 19.0 | 18.2 | 21.7 | 19.9 | 19.4 | 21.1 | 18.9 | 18.1 | **22.6** | 20.1 | 33.4 |
| ***Medium municipalities (n = 334)*** |  |  |  |  |  |  |  |  |  |  |  |  |
| Sensitivity (%) | 85.2 | 92.8 | **93.9** | 83.7 | 92.8 | 93.6 | 89.4 | 92.8 | 93.6 | 87.5 | 91.7 | 90.3 |
| Timeliness (%) | 80.8 | 91.9 | **91.7** | 82.3 | 89.6 | 90.5 | 87.6 | 90.4 | 90.5 | 86.6 | 88.8 | 78.4 |
| PPV (%) | 17.9 | 14.1 | 13.1 | 19.9 | 16.1 | 15.0 | 17.9 | 13.5 | 12.6 | **20.8** | 16.2 | 28.1 |
| **Large municipalities (n = 316)** |  |  |  |  |  |  |  |  |  |  |  |  |
| Sensitivity (%) | 84.1 | 93.0 | 93.8 | 84.5 | 93.0 | 93.0 | 90.7 | 93.8 | **93.8** | 88.8 | 92.6 | 92.6 |
| Timeliness (%) | 83.3 | 93.0 | 94.3 | 83.0 | 95.1 | 95.1 | 89.2 | 93.9 | **95.1** | 88.2 | 94.7 | 94.7 |
| PPV (%) | 18.9 | 15.9 | 14.8 | 21.0 | 17.2 | 15.9 | 19.5 | 15.7 | 14.7 | **20.9** | 18.0 | 17.4 |

**References**

1 Hoehle M, Meyer S, Paul M. surveillance: Temporal and Spatio-Temporal Modeling and Monitoring of Epidemic Phenomena. CRAN: Contributed Packages. 2024; **52**: 4357–4368.

2 Craig AT, Leong RNF, Donoghoe MW, Muscatello D, Mojica VJC, Octavo CJM. Comparison of statistical methods for the early detection of disease outbreaks in small population settings. *IJID Regions* 2023; **8**: 157–163.

3 Hutwagner L, Thompson W, Seeman GM, Treadwell T. The bioterrorism preparedness and response Early Aberration Reporting System (EARS). *Journal of Urban Health* 2003; **80**: i89–i96.
